# Opportunities for Orphan Crops: Expected Economic Benefits From Biotechnology

**DOI:** 10.3389/fpls.2022.825930

**Published:** 2022-06-23

**Authors:** Patricia Zambrano, Ulrike Wood-Sichra, Remidius D. Ruhinduka, Dayo Phillip, Alejandro Nin Pratt, John Komen, Enoch Mutebi Kikulwe, José Falck Zepeda, Fred M. Dzanku, Judith A. Chambers

**Affiliations:** ^1^International Food Policy Research Institute, Washington, DC, United States; ^2^Independent Researcher, Krems, Austria; ^3^School of Economics, University of Dar es Salaam, Dar es Salaam, Tanzania; ^4^Centre for Agriculture and Rural Development Studies, Federal University of Lafia, Lafia Nigeria; ^5^Komen Bioscience Consultancy, Haarlem, Netherlands; ^6^Alliance of Bioversity International and CIAT, Kampala, Uganda; ^7^Institute of Statistical, Social and Economic Research, University of Ghana, Accra, Ghana

**Keywords:** GMO crops, impact assessment, economic surplus model, DREAMpy, sub-Sahara Africa, regulatory policy

## Abstract

An enabling, evidence-based decision-making framework is critical to support agricultural biotechnology innovation, and to ensure farmers’ access to genetically modified (GM) crops, including orphan crop varieties. A key element, and often a challenge in the decision-making process, involves the balancing of identified potential risks with expected economic benefits from GM crops. The latter is particularly challenging in the case of orphan crops, for which solid economic data is scarce. To address this challenge, the International Food Policy Research Institute (IFPRI) in collaboration with local economists analyzed the expected economic benefits to farmers and consumers from the adoption of GM crops in 5 sub-Saharan African countries. This paper focuses on case studies involving insect-resistant cowpea in Nigeria and Ghana; disease-resistant cassava in Uganda and Tanzania; and disease-resistant banana in Uganda. Estimations from these case studies show substantial economic benefits to farmers and consumers from the timely adoption and planting in farmers’ fields of GM orphan crops. Our analysis also shows how the benefits would significantly be reduced by regulatory or other delays that affect the timely release of these crops. These findings underscore the importance of having an enabling policy environment and regulatory system—covering, among other elements, biosafety and food/feed safety assessment, and varietal release registration—that is efficient, predictable, and transparent to ensure that the projected economic benefits are delivered and realized in a timely manner.

## Introduction

Despite their critical local importance, orphan, underutilized, and neglected crops tend to receive relatively limited attention in agricultural R&D initiatives (Tadele, 2019). While these crops play a key role in local diets and are uniquely adapted to the environment in which they are grown, they are characterized by underfunding for research and development, very little attention from agriculture extension services, weak and underdeveloped value chains, lack of awareness about their nutritional value, a perception that they are a “poor farmer’s crop” and low interest among farmers and industry due to lack of demand. Additionally, many orphan crops have large and complex genomes, which has limited the success of conventional research approaches. However, in recent years, genetic modification (GM) has been successfully applied to address some key production constraints faced by orphan crops. This has increased interest in the potential of GM orphan crops to boost local food security and agri-business as evidenced by a growing R&D and field-testing pipeline as presented in this article.

The cultivation of GM crops has increased over the years from just a handful of countries in 1996—the United States, Argentina, Canada, Australia, and Mexico—to 29 adopting countries in 2019 (ISAAA, 2019). Commercial cultivation of GM crops is still largely confined to maize, soybeans, cotton, and canola, with no representation of orphan crops in SSA. This situation changed with the approval of a GM cowpea variety SAMPEA 20-T by Nigeria’s National Committee on Naming, Registration and Release of Crop Varieties. This local GM cowpea variety expresses resistance to the pod borer *Maruca vitrata* (Okojie, 2019).

Considering a 25-year track record of safe cultivation and consumption, and a growing body of literature assessing the economic benefits of GM crops (National Academies of Sciences, Engineering, and Medicine (NASEM), 2016), countries in SSA have continued to steadily expand their GM crops R&D pipeline. Until 2012, the area planted to GM crops on the continent was limited to South Africa. USDA-FAS (2020) reports that South Africa currently cultivates GM maize, soybeans and cotton, with very high GM adoption rates for all three crops (82, 95, and 100 percent, respectively). In the 2020–2021 production season, a total of 2.9 million hectares of GM crops were planted in South Africa. GM maize plantings represent about 75 percent of total GM crop plantings in South Africa, followed by soybeans (24 percent), and GM cotton representing around 1 percent (USDA-FAS, 2020). Most of the GM maize (over 70 percent) under cultivation in South Africa involves “stacked” improvements, combining insect resistance and herbicide tolerance (USDA-FAS, 2020).

Outside of South Africa, the momentum in African economies towards authorizing the cultivation of GM crops appears to be building up. In 2019, the Kingdom of Eswatini (formerly Swaziland) joined South Africa and Sudan in planting GM crops, with commercial planting of insect resistant Bt cotton. In that same year Nigeria, Ethiopia, Kenya, and Malawi granted approvals for planting GM cotton. Approval for GM cotton in Nigeria was followed by the commercial registration of insect resistant cowpea as mentioned above, and, in 2021 the Government of Nigeria authorized the general environmental release of “TELA maize,” which is tolerant to insect pests and drought (Premium Times, 2021).

Table 1 presents the current area planted to GM crops in SSA. For a more detailed overview, Akinbo et al. (2021) present and analyze the status of GM crop adoption in Sub-Sahara Africa (SSA), including an overview of GM crop confined field trials.

**Table 1:** GM crop cultivation in sub-Saharan Africa.

Country	GM crop/trait	Area planted (hectares)	Year
Eswatini	Insect-resistant cotton	403	2019
Ethiopia	Insect-resistant cotton	311	2019
Kenya	Insect-resistant cotton	n.a.	2020
Malawi	Insect-resistant cotton	6,000	2019
Nigeria	Insect-resistant cotton	700	2019
	Insect-resistant cowpea	n.a.	2021
South Africa	Insect-resistant, herbicide-tolerant maize	2,134,000	2020
	Herbicide-tolerant soybean	785,745	2020
	Insect-resistant cotton	16,176	2020
Sudan	Insect-resistant cotton	236,200	2019

Source: ISAAA (2021); figures for South Africa: USDA-FAS (2020).

n.a., data not yet available.

Like other adopting countries most of the countries listed in Table 1 have authorized GM crop cultivation after implementing regulatory frameworks that include food, feed, and environmental safety assessments, which follow international safety assessment standards. Numerous countries, including some in Africa, have authorized GM crop field trials and cultivation after implementing regulatory frameworks, and have also taken into consideration the potential economic benefits and costs of GM crops’ adoption and use, such as their impact on trade, farmers and consumers. However, even though the number of peer-reviewed publications analyzing the economic impacts of GM crop cultivation in SSA has substantially increased over the last 15 years, the majority of articles are still focused on cotton and maize in South Africa (Zambrano et al., 2019). Additionally, the number of GM crops’ economic assessment studies that are generated in-country are few; a fact that is often cited as a hurdle in the adoption of GM crops. In this article, we address this gap by focusing on the potential benefits GM orphan crops in in Ghana, Nigeria, Tanzania, and Uganda.

To address the gap identified above, the International Food Policy Research Institute (IFPRI) jointly with in-country economists, designed and implemented a project, titled, “Biotechnology and Biosafety Rapid Assessment and Policy Platform” (BioRAPP). Unlike many of the previous ex ante or predictive studies on GM crops, BioRAPP was purposely designed to assure the active participation of relevant policy and decision makers as well as other local stakeholders, whose viewpoints were not just considered, but determined the selection of the specific crops of interest to each of their countries, provided guidance throughout the project and selected local economists who led each country study. In other words, BioRAPP’s starting point was an extensive consultation process in each of the countries where the ex-ante evaluations were performed.

In the next two sections we present the project’s overall methodology and research findings for the ex-ante assessments of banana, cassava, and cowpea, the orphan crops included in the BioRAPP project. The final section discusses the importance of these type of studies for decision makers, who need access to current and robust knowledge about potential impacts and tradeoffs to support innovation that may help address pressing agricultural constraints.

## Materials and Methods

Overall, the BioRAPP project produced a total of 8 GM crop/trait ex ante case studies led by in-country economists in Ethiopia, Ghana, Nigeria, Tanzania, and Uganda (Dzanku et al., 2018; Phillip et al., 2019; Kikulwe et al., 2020; Ruhinduka et al., 2020; Yirga et al., 2020, respectively). Additionally, Zambrano et al. (2019) published an overview of the economic assessment literature for Africa and an updated web bibliography on the economic assessment of GM crops (bEcon). Table 2 lists all 8 BioRAPP ex ante assessments, five of which were focused on cassava, cowpea, and banana, which can be classified as orphan crops and are the subject of this article.

**Table 2:** BioRAPP ex ante case-study assessments.

Country	Trait	Orphan crop	Non-orphan crop
Ethiopia	Water efficient and stemborer resistant		Maize
Ghana	Nitrogen and water efficient, salt tolerant		Rice
	Pod borer resistant	*Cowpea*	
Nigeria	Pod borer resistant	*Cowpea*	
Tanzania	Brown streak disease resistant	*Cassava*	
	Water efficient and stemborer resistant		Maize
Uganda	Brown streak disease resistant	*Cassava*	
	Bacterial wilt disease resistant	*Banana*	

Source: Dzanku et al. (2018), Phillip et al. (2019), Kikulwe et al. (2020), Ruhinduka et al. (2020), and Yirga et al. (2020).

### Case Study Selection and Project Setup

The GM crops listed in Table 2 are at various stages of technology progress. In Nigeria, the confined field tests for GM pod borer-resistant cowpea (PBR cowpea) started in 2009 and have steadily progressed towards biosafety authorization for general environmental release, food and feed safety clearance, resulting in registration as a commercial variety in December 2019. Prior to its release for cultivation by farmers, PBR cowpea was selected as a case study under BioRAPP. In Ghana, PBR cowpea has gone through multi-location confined field trials and currently (April 2022) awaits biosafety review for general environmental release.

In the case of disease-resistant cassava in Uganda, after a decade of research and safety testing by the National Agricultural Research Organization (NARO) and partners, GM virus-resistant cassava approached a stage where it can be released to farmers, contingent upon final biosafety clearance and variety release procedures by relevant authorities. To date, Ugandan authorities have assessed the safety and efficacy of GM cassava at multiple stages, including laboratory, greenhouse and multi-location confined field trial stages (since 2010). In Tanzania, local authorities tested the GM cassava under greenhouse conditions in 2017.

Lastly, in Uganda NARO started GM banana improvement in the early 2000s. A major focus for this work involved approaches to achieve resistance to Banana Xanthomas Wilt (BXW), a disease that is currently having devastating effects. NARO initiated confined field trials for BXW-resistant bananas in 2010 followed by multi-location trials in 2016 using the best performing lines.

For the case-study analysis in each country, BioRAPP’s initial and most important efforts were aimed at assuring input by local, relevant stakeholders and experts. Once these stakeholders were identified, meetings were held to discuss which GM crops to select for the case studies, to identify the implementing local agencies and partnerships and the selection of local lead economists. The case study selection was finalized in consultation with policymakers, regulators, and crop scientists and was based on: the stage of product development (beyond proof of concept and approaching general release); the crop’s economic and social relevance; and the potential for these assessments to help inform policy and regulatory reforms.

Following the initial consultations, BioRAPP established collaborative agreements with local partner organizations and lead economists, and appointed local steering committees to provide guidance and expert review of preliminary findings throughout the project’s duration. Lead economists collected secondary data and elicited key expert opinions. Data sources included:

- National and regional crop production statistics such as area planted, yield, crop prices, demand, and growth rates.
- Available household and farm-level survey data.
- Crop use and costs of inputs such as seed, fertilizer, pesticides, and labor.
- Adoption rates of comparable improved varieties.

The projected estimates of some key parameters rely on local experts’ informed opinions. Interviews and consultation meetings were held to elicit their expert opinions on parameters influencing costs and benefits, such as technology adoption rates, expected yield changes, and delivery timelines. Panel data thus generated were analyzed by teams of local economists in collaboration with IFPRI economists using the analytical approach summarized in the next section.

### Analytical Approach: Economic Surplus Model^1^

The estimations presented in this article are based on a multi-region economic surplus model (ESM; Alston et al., 1995). This model has been used extensively over the years in multiple countries to evaluate and prioritize research investments. Specifically for the evaluation of GM crops in developing economies, Smale et al. (2009) comprehensive research methodology on GM crops documented nine peer-reviewed papers that apply the ESM, implemented for Argentina, Colombia, China, India, Kenya, Mexico and Uruguay.

Smale et al. (2009) and Petsakos et al. (2018) have indicated that the ESM has important limitations given that it does not explicitly consider market failures, input markets, transaction costs, externalities, and general equilibrium effects. Nevertheless, the ESM as implemented in the BioRAPP project has a critical advantage over other more comprehensive methods because it is parsimonious in data requirements and it is, in comparison to other methods, easy to understand, learn and implement particularly when facing data and time constraints.

**Figure 1:**
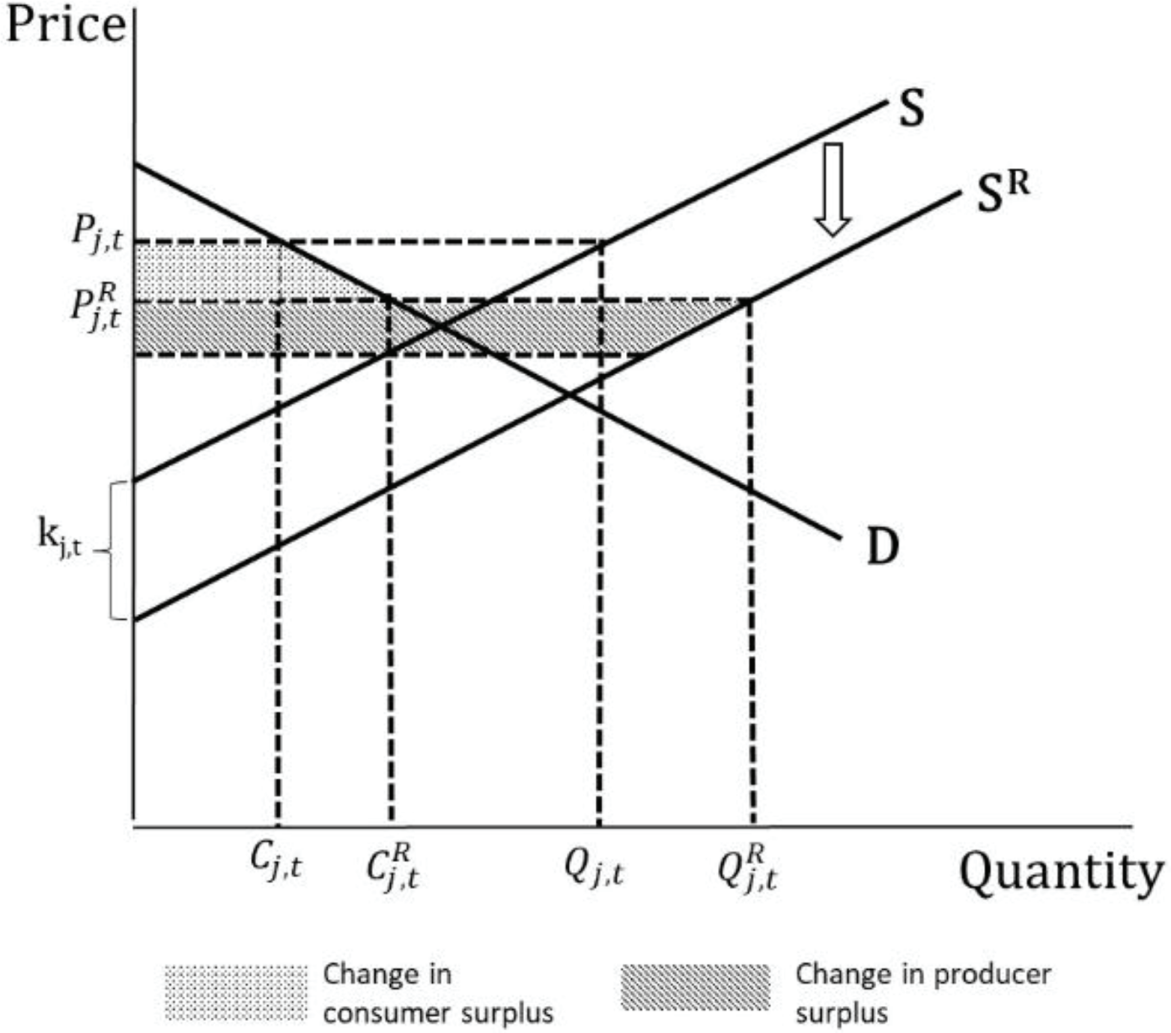
Size and distribution of research benefits. *Source*: Authors’ elaboration.


Following Alston et al. (1995), Figure 1 shows changes arising from research driven supply shift for country/region *j* and time *t* in the horizontal multi-market model for a single product. The initial supply curve is S, and demand D with a market clearing price $P_{j,t}$ in country/region *j* and time *t*. Supply curve S shifts downward and rightward to S^R^, with a new market clearing regional price $P_{j.t}^{R}$ . The shaded areas in Figure 1 represent the change in producer and consumer surplus. One can derive algebraically the shaded areas in Figure 1, yielding the following equations to examine economic welfare effects:

(1) $$\begin{array}{c} \Delta PS_{j,t}=\left(k_{j.t}+PP_{j.t}^{R}-PP_{j,t}\right)\left[Q_{\left(j,t\right)}+0.5\left(Q_{j,t}^{R}-Q_{j,t}\right)\right]\;\\ \;\mathrm{Change}\;\mathrm{in}\;\mathrm{Producer}\;\mathrm{Surplus} \end{array}$$

(2) $$\begin{array}{c} \Delta CS_{j,t}=\left(PC_{j,t}-PC_{j,t}^{R}\right)\left[C_{j,t}+0.5\left(C_{j,t}^{R}-C_{j,t}\right)\right]\;\\ \;\mathrm{Change}\;\mathrm{in}\;\mathrm{Consumer}\;\mathrm{Surplus} \end{array}$$

where Δ $PS_{j,t}$ is change in producer research benefits and Δ $CS_{j,t}$ is change in consumer research benefits in region *j* in a given year *t*. In turn, $Q_{j,t}$ , $C_{j,t}$ , $PP_{j,t}$ , $PC_{j,t}$ are initial production, consumption, producer prices, and consumer prices respectively, in country/region *j* and time *t*. Furthermore, $C_{j,t}^{R}$ , $Q_{j,t}^{R}$ , $PP_{j,t}^{R}$ and $PC_{j,t}^{R}$ are new consumption, production, producer price, and consumer price in country/region *j* and time *t*, with research. These general equations are manipulated and translated to elasticity form to derive estimating equations using available parameters in the literature, operationalized in IFPRI’s Dynamic Research Evaluation for Management, Python version (DREAMpy) software (IFPRI, 2019). The DREAMpy approach is a user-friendly open-source software, which follows Alston et al. (1995, p. 212–218) formulation of a multi-region technology adoption with associated production characteristics.

The study results were presented using alternative scenarios based on varying assumptions, of three key parameters: (1) potential yield gain from the adoption of GM varieties; (2) potential adoption rates; and (3) expected change in production cost due to adoption of GM varieties considering alternative estimated changes in seed and input costs. Case studies used minimum, most likely, and maximum values for these three parameters to derive multiple scenarios. Developing multiple scenarios is a standard procedure when performing sensitivity analyses in economic impact evaluations.

## Results: Case Study Synthesis

Applying the ESM approach summarized above, the overall estimations confirm significant economic benefits from adopting GM orphan crops. Table 3 presents the estimated total net benefits (Net Present Value) for the GM orphan crops selected as focus for this article. While the main figures reflect the most likely scenario, the range of simulation outcomes under different scenarios (as described in “Materials and Methods” above) is included as well.

**Table 3:** Estimated present value of producers and consumers, and total net present value from adopting GM orphan crops by country (millions of US$).

Country	GM crop	Base year	Present value and net present value^*^ *(Min–max range)*		
			Producers	Consumers	Total
Ghana	Pod borer resistant cowpea	2014	8.6 *(3.2–54)*	12 *(4.5–73)*	19 *(5.6–125)*
Nigeria	Pod borer resistant cowpea	2016	258 *(14–781*)	91 (*4.7–316)*	336 *(10.4–1,081*)
Tanzania	Brown streak disease resistant cassava	2015	65 *(7.5–159)*	85 *(8.6–221)*	150 *(14–377)*
Uganda	Brown streak disease resistant cassava	2015	90 *(−16–254)*	481 *(366–614)*	570 *(409–750)*
Uganda	Bacterial wilt disease resistant banana	2015	326 *(22–632)*	459 *(291–679)*	775 *(499–1,101)*

Source: Dzanku et al. (2018), Phillip et al. (2019), Kikulwe et al. (2020), and Ruhinduka et al. (2020).

* Figures correspond to most likely values, while those in parentheses and italics to minimum and maximum values. Estimations were done on local currency units and converted to US dollars using the official annual exchange rate for the base year listed.

### Pod Borer-Resistant Cowpea in Ghana and Nigeria

Based on secondary data analysis and expert consultations, the estimated net present value (NPV) of adopting the PBR cowpea in Ghana is US$ 19 million. Simulations using a range of values for key parameters indicate that this value could fluctuate between US$ 5.6 million and US$ 125 million. As described above, these simulations were constructed based on literature review (e.g., publications on the adoption of improved, conventional varieties), the use of household survey data, and expert informed opinions regarding the expected changes in cowpea yield, production cost (e.g., savings in insecticide applications), and expected adoption rates.

In comparison to Ghana, the expected benefits from adopting PBR cowpea are substantially higher in Nigeria, the world leader in cowpea production (Box 1). The estimated present value of the net benefits of adopting PBR cowpea amount to US$ 336 million, of which more than 75 percent is accrued by producers with the balance accrued by consumers. Given that actual planting of PBR cowpea started in June 2021, it will be now possible to assess the realized benefits of this GM crop in a few years. Anecdotal evidence of the first year of adoption appear to confirm that farmers gain from the adoption of PBR cowpea, with self-reported yield increase and decreased pesticide application (Opoku Gakpo, 2021).

Box 1 **Cowpea’s economic importance**

Cowpea is grown in tropical Africa, Asia, North and South America mostly as a grain, but also as a vegetable and fodder crop. It is favored because of its wide adaptation and tolerance to several stresses. It is an important food source and is estimated to be the major protein source for more than 200 million people in sub-Saharan Africa (OECD, 2015). Cowpea is the most important grain legume crop in Nigeria and West Africa. It is a key cash crop and plays a critical nutritional and food security role as a source of cheap protein and animal feed and as a source of cash, while also contributing to soil fertility improvement. Nigeria is the largest African producer, followed by Niger, Burkina Faso, Cameroon, and Tanzania. In 2015, Nigeria produced an estimated 2.2 million metric tons of cowpea and consumed 2.6 million metric tons. Cowpea is targeted by multiple insect pests and diseases from planting to storage. Postflowering insect pests such as the legume pod borer, flower thrips and pod-sucking bugs can cause grain yield losses from 55 to 100 percent if not controlled.

*Source*: Phillip et al. (2019).

**Figure 2:**
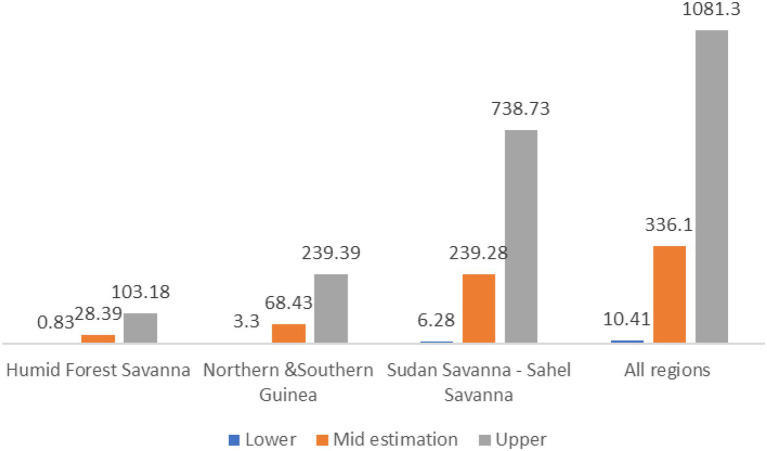
PBR cowpea in Nigeria: Net Present Benefits (NPV) over a 25-year period if PBR cowpea is planted in 2020 (million US$; Phillip et al., 2019). Estimations were done in local currency units, converted to US dollars using the official annual exchange rate for 2016, the base year used for these estimations.

BioRAPP data analysis allows differentiation of impacts across national geographical areas. Figure 2 illustrates the case of PBR cowpea benefit distribution for three different areas in Nigeria. Such regional estimations can be useful for policymakers who wish to better target investments. In the case of PBR cowpea in Nigeria, a large share of net present benefits will accrue to the Sudan–Sahel savannah region, where 40 percent of all cowpea production is concentrated, and where the adoption rate and the maximum induced change in cowpea yields is projected to be realized. On the other end is the forest region, with the lowest estimated adoption rates and induced yield. Given that the forest region produces only 20 percent of cowpea but demands 40 percent of all cowpeas traded in Nigeria, the expected benefits are mostly gained by the induced decrease in price that will benefit consumers in the region. While the distribution of benefits across countries will be different, regional differences are also observed in Ghana.

### Brown Streak Disease Resistant Cassava (Uganda, Tanzania)

The results also show significant regional differences from the adoption GM cassava in Uganda due to the expected regional variance in adoption rates for new cassava technologies. Considering the importance of cassava in Uganda, as well as other countries in East Africa (Box 2), the total benefits to producers and consumers from adopting GM, virus-resistant cassava are an average of US$ 18.4 million per year, over the 31 years of the economic model simulation. Net benefits varied from US$ 1 million/year in the Western region to US$ 8.7 million/year in the Eastern region. In Tanzania, the expected total net benefits range from US$ 15 million/year under an optimistic scenario to US$ 0.5 million/year under a pessimistic scenario.

Box 2 **Cassava viral diseases in East Africa**

Cassava serves as a primary source of calories for more than 250 million people living in sub-Sahara Africa. It grows well in poor soils with limited rainfall. Cassava production in East Africa, including Uganda and Tanzania, is hampered by two yield-reducing viral diseases—namely, the cassava brown streak disease (CBSD) commonly referred to as the “Ebola of plants” and cassava mosaic disease (CMD). Particularly, CBSD can be responsible for a complete loss of harvest. Addressing the two diseases would enhance cassava farmers’ ability to capture additional income through new business opportunities, while opening alternative value-chain channels through cassava industrialization.

*Source*: Kikulwe et al. (2020), and Ruhinduka et al. (2020).

### Bacterial Wilt Disease Resistant Banana (Uganda)

Estimations for GM, BXW-resistant banana show substantial potential benefits to farmers and consumers from improving resistance to a devastating disease (Box 3). Average annual benefits are estimated at US$ 25 million, of which producers receive US$15 million and consumers US$ 10 million. The average annual benefits per hectare are US$ 293.

Box 3 **The banana *Xanthomas* wilt challenge in Uganda**

In 2018 Uganda had the second largest area harvested with bananas in Africa with 21 percent of the total area under banana cultivation across Africa and 16 percent of production. Banana production in Uganda amounted to 4.6 million metric tons in 2014, of which 3,070 metric tons were exported. Banana yields reached a peak 5.33 tons/ha in 1994 but is decreasing steadily since then, down to 4.6 tons/ha in 2018. This decrease cannot be attributed solely to plant pathogens such as bacterial wilt. Other reasons for the observed yield decrease include soil fertility declines, pests (nematodes and weevils) and moisture stresses, and institutional and market constraints such as input access and low prices. The area harvested increased from 1961 through 2007 reaching a peak of 1.8 million hectares. Area harvested was just over 1 million hectares in 2018. Total production increased from 6 million tons in 1980 to 10.5 million tons in 2002, declining to 4.3 million tons in 2018. Bananas are the most widely consumed food in Uganda, making them critical to food and income security for families. The average Ugandan eats 220 kg of bananas a year, and for 66 percent of the country’s urban population, bananas are a staple food. Bananas are mainly produced by smallholder farmers. Overall, more than 75 percent of all farmers in Uganda grow bananas.

Banana Xanthomonas Wilt (BXW), first report in Uganda in 2001, affects all banana types. National experts estimated that BXW could destroy 90 percent of all bananas in Uganda if not controlled. BXW can completely decimate individual banana plots and thus heavily impact farmer’s food security. To address BXW’s devastating impacts on farmers, the Ugandan government invested heavily in controlling BXW using agronomic and cultural practices. The recommended control methods included destruction and disposal of infected plants, disinfecting tools used in the plantation, using clean planting material and removing male buds. Such measures are costly and only effective when implemented rigorously and must be complemented by crop improvement programs to generate resistant varieties. And, despite these efforts, the emergence of BXW in Uganda continues to pose a significant problem.

*Source*: Kikulwe et al. (2020).

Using well-established methods to estimate changes in poverty status derived from changes in income (Moyo et al., 2007; Alene et al., 2018), the BioRAPP analysis confirms that the additional economic income created by deploying BXW-resistant banana would be the equivalent of 54,776 Ugandans escaping poverty each year. Similarly, benefits from growing a CBSD-resistant cassava would be the equivalent of 40,308 Ugandans escaping poverty each year. The two disease-protected crops would have a sizable impact on Ugandan’s livelihoods.

### Investing in GM Orphan Crops Yields Positive Returns

**Figure 3:**
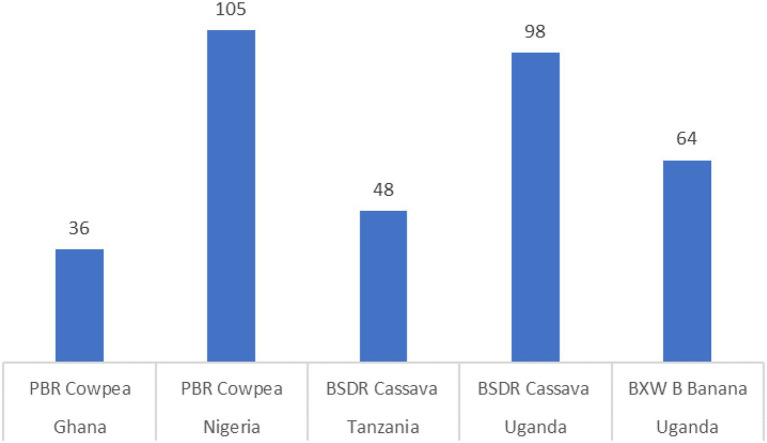
Internal rate of return (IRR)^*^ to R&D investments incurred in-country by crop/trait (percentages; Dzanku et al., 2018; Phillip et al., 2019; Kikulwe et al., 2020; Ruhinduka et al., 2020). ^*^IRR estimates include only R&D costs incurred in country.

Figure 3 introduces estimates for the internal rate of return (IRR) derived from the adoption of GM orphan crops in the five case study countries described in this article. This is a key indicator estimating the economic profitability of a proposed R&D investment incurred in-country. The in-country cost estimates in our case studies consider the local R&D costs of adapting the technology to the recipient environment, and the required expenses to secure regulatory compliance, seed registration and in some cases technology deployment and product stewardship. It is important to note that R&D costs by developers, as they are incurred as part of international collaborative research programs, were not included in the R&D cost estimates. Obviously, this results in higher IRRs compared to those including all R&D costs; however, in this case, they are a valuable indicator to guide local investment decisions. Figure 3 presents the IRR under the most likely scenario—regarding expected adoption rates, yield increases. and production cost reductions.

Given the relatively low R&D costs, the expected IRR of GM investments is high—estimated to reach a high of 105 percent in the case of PBR cowpea in Nigeria and a minimum of 36 percent for PBR cowpea in Ghana. As explained above, the IRRs presented in Figure 3 contrast with the 40–60 percent range typically found in agricultural R&D projects (Alston et al., 2000). Including local and international costs of R&D will likely bring our findings in line with these averages.

### Delays in Research, Decision-Making, and Adoption Reduce the Rate of Return

**Figure 4:**
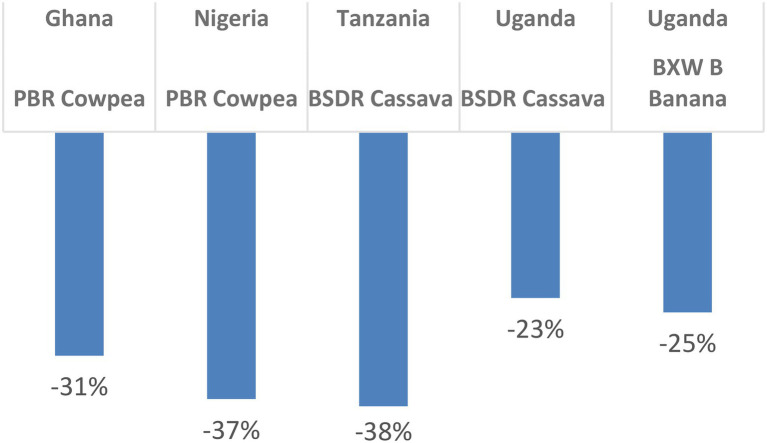
Economic losses due to a 5-year delay^*^ during the R&D/regulatory stages (Dzanku et al., 2018; Phillip et al., 2019; Kikulwe et al., 2020; Ruhinduka et al., 2020). ^*^Impacts of the cost of regulatory delays are estimated maintaining constant the number of years for the simulation after shocking the cash flows with a 5-year delay.

Several studies (see for example Falck Zepeda et al., 2008a,b; Smyth et al., 2014; Wesseler et al., 2017) point to the economic impact of time delays in GM adoption resulting in foregone benefits. In the BioRAPP study, a 5-year adoption delay was simulated due to possible slowdown in the R&D process, in regulatory decision-making and/or in actual technology adoption (e.g., due to delays in seed availability). Such delays have significant impact on outcomes. An overview of expected economic losses due to a 5-year time delay is presented in Figure 4 below, ranging from 23 percent in the case of brown streak disease-resistant (BSDR) cassava in Uganda to 38 percent in the case of BSDR cassava in Tanzania.

A specific example of the effect of time delays for Uganda, show that a 5-year delay in the research and regulatory process for BSDR cassava reduces the rate of return from 98 to 36 percent, whereas a 5-year delay during the adoption process reduces rate of return to 46 percent. In the case of BXW-resistant bananas in Uganda, a 5-year research and regulatory delay reduces the rate of return from 98 to 60 percent whereas a 5-year adoption delay reduces this rate to 85 percent. The timely variety registration of PBR cowpea in Nigeria, followed by on-farm demonstrations and seed multiplication in 2020 and commercial launch in 2021, will help minimize this potential loss in benefits.

## Discussion

The case study analysis presented above adds value to the existing literature on the economic assessment of GM crops as it expands the analysis from cotton and maize to orphan, food security crops in countries where similar estimations are scant. In addition, the BioRAPP project went beyond the customary desktop study to help build a foundation for future assessments by ensuring ownership by local teams, crop experts and economists. The unique nature of the project design and implementation contributed to its impact on building local capacities: Formal government buy-in from each country was the first intermediate outcome that was necessary to achieve before the economic assessments started. This was followed by identifying the appropriate local institutions, crop experts, and economists to lead the ex-ante assessments, and the formation of multi-stakeholder steering committees whose members helped determine the crop/trait case study selections and contributed critical inputs. The analytical tool developed as part of the project, DREAMpy, allows for the generation of rapid but robust estimates of economic costs and benefits of R&D projects, which is now being used beyond the purpose of this study.

The case study analysis confirms the important, expected economic benefits to materialize from the adoption of GM crops in SSA. Considering the substantial positive internal rates of return, investing in the development of GM orphan crops and their adoption is a worthwhile proposition for decision makers in the case-study countries and for SSA generally. The case studies confirm overall findings from previous ex ante and ex post economic analysis in SSA, as documented in the bEcon 4 Africa literature review by Zambrano et al. (2019), notwithstanding the significant variability across crops, countries, and regions.

Obviously, this type of predictive, ex ante analysis is not without limitations. For the case study analysis, assessing the impacts of the introduction of a crop technology requires two types of data. First, a set of acceptable crop production and consumption related data and, second, a set of robust assumptions about how the introduction of the technology will affect yields and crop management. As the BioRAPP analysis primarily relied on existing, secondary data, a major hurdle lies in the fact that such secondary data are rarely readily available, comprehensive, comparable or even reliable. Local research teams invested substantial effort to collect and analyze relevant data from a wide variety of sources and locations. Assumptions, which determine the size and direction of the estimated economic benefits, are hardly found in formal publications. Establishing robust scenarios and assumptions demanded a series of expert consultations, which in turn had to be triangulated against published and unpublished data. Additional, ex post analysis following the actual adoption of GM crops (e.g., PBR cowpea in Nigeria) will be required to validate the main findings from this work.

The results highlight the value of participatory economic and social assessments in support of decision making, as decision makers need to carefully consider which socioeconomic issues and assessments may be feasible for inclusion in a regulatory process.

Case study results also confirmed the fact that delays, particularly in the R&D process and in regulatory review processes, rather than the later adoption stages, significantly reduce anticipated benefits. These findings underscore the opportunity for policymakers and decision makers to invest in policies and programs that affect these critical variables, particularly to foster conditions to make available these crops to farmers and consumers. Investment in effective extension practices and seed systems are critical factors that merit the attention of decision makers. Value chain, seed systems, and market intelligence analysis which target products for profiling prior to release, can be critical to deployment of GM-based crop improvement.

Given that GM crops are regulated, it is critical that a competent, efficient biosafety regulatory system is in place to ensure that improved varieties reach farmers in a timely manner and thus allow the realization of expected economic benefits (Adenle et al., 2018). The crop/trait cases assessed in this study are unlikely to be deployed without an enabling regulatory environment for biosafety review, food and feed safety assessment and subsequent variety registration. As described in this paper, financial costs of regulatory compliance are unlikely to reduce the internal rates of return significantly, especially when evaluated against the potential economic gains. Financial cost may, however, be a limiting factor for the public sector who usually faces budget constraints and in countries where the resources to conduct robust economic assessments may be limited. Nevertheless, time delays due to R&D and/or regulatory compliance issues are the factors that decrease rates of return significantly. Therefore, avoiding unnecessary delays are a major objective for public policy, which will require attention from policy and decision makers, as well as regulators and other stakeholders, especially for public sector R&D (Smyth et al., 2014).

While regulatory policy is not singular in terms of its impact on the adoption of innovations, it is an important component that must be critically evaluated in connection with GM crops. Ultimately, an evidence-based, efficient, predictable and transparent regulatory system, which is well understood by stakeholders and well implemented, can potentially lead to valuable, safe and appropriate GM crops in the hands of farmers.

## Conclusion

As the area planted to GM crops continues to grow in SSA, the interest in, and the need for economic benefit assessment along with safety assessment are most likely to increase. The research methodology, tools and case studies developed under BioRAPP and presented in this article show that such assessments can be conducted by local experts and stakeholders and can yield relatively rapid assessments for consideration or demanded by decision makers. While recognizing that further work is required, as such, our findings support statements included in the recent “Africa Common Position to the UN Food Systems Summit” (African Union, 2021), which emphasizes that:

*“[…] Increased support for the adoption of biotechnology, particularly among smallholders, including the new generation of farmers emerging across Africa, requires accelerated action and a conducive enabling environment. Scientists are designing and developing livestock breeds and crop varieties with higher yields, additional nutrients (*e.g.*, in biofortified crop seeds, roots and cultivars), increased disease resistance and enhanced tastes through crop biotechnology and genomics. The power of modern agricultural biotechnology and genomics in transforming African food systems into a force of economic growth, creating wealth in the rural space and beyond, feeding an African population expected to reach 2.2 billion people by 2050 cannot be ignored. […] Despite challenges and uncertainties surrounding the adoption of biotechnology adoption, there appears to be a significant potential for capturing large economic, social, and environmental payoffs from the use of biotechnology products in the farming systems across Africa.”* (African Union, 2021; p. 8)

## Author Contributions

JK conceived the overall contents and structure for this article. JK, PZ, JZ, and JC wrote and reviewed successive drafts. PZ, UW-S, JZ, and AP led the data analysis underpinning the BioRAPP project. UW-S, PZ, and JZ drafted tables and performed analysis from data collected for each of the country studies. RR, DP, EK, and FD contributed to this article as PI’s of their respective BioRAPP country studies and as lead authors of discussion papers referenced below. AP, PZ, UW-S, and JZ gave analytical and modelling support to all PI’s during the development of each country study. All authors contributed to the article and approved the submitted version.

## Funding

BioRAPP was a project co-funded by the Bill and Melinda Gates Foundation (grant number INV-009513/OPP1131119) and the US Agency for International Development (award number AID-BFS-IO-14-00001), supported by the CGIAR Research Program on Policies, Institutions, and Markets (PIM), led by IFPRI, and funded by CGIAR Fund Donors.

## Conflict of Interest

JK was employed by Komen Bioscience Consultancy.

The remaining authors declare that the research was conducted in the absence of any commercial or financial relationships that could be construed as a potential conflict of interest.

## Publisher’s Note

All claims expressed in this article are solely those of the authors and do not necessarily represent those of their affiliated organizations, or those of the publisher, the editors and the reviewers. Any product that may be evaluated in this article, or claim that may be made by its manufacturer, is not guaranteed or endorsed by the publisher.

## Footnotes

^1^For a detailed review of the analytical approach, methodology, and assumptions, we refer to the IFPRI Discussion Papers listed in the references section (Dzanku et al., 2018; Phillip et al., 2019; Kikulwe et al., 2020; Ruhinduka et al., 2020).
